# Withaferin A inhibits Epithelial to Mesenchymal Transition in Non-Small Cell Lung Cancer Cells

**DOI:** 10.1038/s41598-018-34018-1

**Published:** 2018-10-24

**Authors:** Al Hassan Kyakulaga, Farrukh Aqil, Radha Munagala, Ramesh C. Gupta

**Affiliations:** 1 Department of Pharmacology and Toxicology, University of Louisville, Louisville, KY 40202 USA; 2 Department of Medicine, University of Louisville, Louisville, KY 40202 USA; 3 James Graham Brown Cancer Center, University of Louisville, Louisville, KY 40202 USA

## Abstract

Lung cancer is the leading cause of cancer-related deaths worldwide and in the United States. Despite recent advancements in treatment approaches, metastasis remains a major therapeutic challenge in lung cancer and explains the extremely poor prognosis. Epithelial to mesenchymal transition (EMT), a complex process of cellular reprogramming has become an attractive drug target because it plays a crucial role in the metastasis of non-small cell lung cancer (NSCLC). In the present study, we examined the effects of withaferin A (WFA), a plant-derived steroidal lactone on EMT in human NSCLC cell lines. First, we demonstrated that WFA displayed time- and concentration-dependent cytotoxicity on A549 and H1299 NSCLC cells. Then, cells were exposed to ≤ 0.5 µM WFA for ≤ 4 h to minimize cytotoxicity and determined its effects on EMT, cell adhesion, motility, migration, and invasion. EMT induction was performed by culturing cells in serum-free media containing TGFβ1 (5 ng/mL) and TNFα (25 ng/mL) for 48 h. We observed that pretreatment of cells with WFA inhibited cell adhesion, migration, and invasion of A549 and H1299 cells. Using western blot, immunofluorescence, and qRT-PCR analysis, we demonstrated that WFA suppressed TGFβ1 and TNFα-induced EMT in both cell lines. Mechanistically, WFA suppressed the phosphorylation and nuclear translocation of Smad2/3 and NF-κB in A549 and H1299 cells. Together, our study provides additional evidence demonstrating the inhibitory effects of WFA on EMT induction in NSCLC cells and further demonstrates the therapeutic potential of WFA against the metastasis in NSCLC.

## Introduction

Lung cancer is the leading cause of cancer-related deaths worldwide^1^ and in the United States^2^. Non-small cell lung cancer (NSCLC) which accounts for about 85–90% of all the lung cancer cases has an overall five-year survival rate of 15–17%^3,4^. Despite the recent advancements in early detection and surgical techniques^5,6^, targeted and immunotherapies^7^, the overall survival from NSCLC has only marginally improved. This extremely poor prognosis is explained in part because about 50–70% of all NSCLC patients are diagnosed when the disease is at an advanced stage and is not curable regardless of treatment approach^5^. Furthermore, the rate of cancer recurrence among NSCLC patients who undergo surgical resection is about 30–70%, most of whom eventually succumb to metastasis^8,9^.

Currently, tumor cell migration, invasion, and metastasis are the main causes of treatment failure and death among NSCLC patients^3,10^. Unlike cellular proliferation, the therapeutic targeting of metastasis has proven difficult and there are no clinically-effective drugs targeting metastasis in NSCLC. This is mainly because metastatic processes are complex, and the underlying mechanisms utilize an interplay of cell adhesion, motility, and survival pathways^11^. Recently, several studies have shown that epithelial-to-mesenchymal transition (EMT), a complex biochemical process of cellular reprogramming plays a crucial role in the metastasis of NSCLC tumor cells^12,13^. During EMT, cells undergo extensive molecular and morphological changes to acquire a mesenchymal phenotype^12^. Normally, EMT is critical in embryogenesis, angiogenesis, and wound healing but tumor cells invoke the EMT process to increase their migratory and invasive capabilities^14,15^. Therefore, given the importance of EMT in metastasis, there has been an increase in the evaluation of small molecule inhibitors of EMT as potential therapeutic drugs against metastasis in NSCLC^11^.

Withaferin-A (WFA) is a biologically-active steroidal lactone that was first isolated from the extracts of the Indian Ayurvedic medicinal plant, *Withania somnifera*^16^. A recent review of available literature on WFA^17^ indicates that WFA displays multiple pharmacological effects including antitumor, anti-inflammatory, pro-apoptotic, immunomodulatory, hepatoprotective, anabolic, antiangiogenic, and anti-fibrotic activities. However, despite these many biological activities, WFA has attracted significant preclinical testing mainly for its antitumor and pro-apoptotic properties. Indeed, several preclinical studies have found WFA to be cytotoxic against lung^18–21^, cervical^22^, prostate^23,24^, breast^25–27^, ovarian^28–30^, colon^31^, B-cell lymphoma^32^, and pancreatic^33^ cancers. More importantly, it has been reported that in addition to its cytotoxic effects, WFA also displays remarkable anti-invasive and anti-metastatic activities^17,27,34^. Although the detailed molecular mechanisms underlying the multiple activities of WFA remain to be fully elucidated, the anti-metastatic effects of WFA have been attributed in part to its inhibition of EMT^17^.

Previously, many studies have reported the anti-EMT properties of WFA on breast^27,34,35^, ovarian^29^, pulmonary^36^, and melanoma^37^ cells. However, data on the underlying mechanisms and the activity of WFA on EMT in NSCLC cells is still limited. In the present study, we examined the effects of WFA on EMT-induction, migration, and invasion in human lung cancer A549 and H1299 cells. Here, we used TGFβ1 and TNFα to experimentally induce EMT, increase motility, migration, and invasion of tumor cells. Our data show that WFA suppressed cytokine-dependent induction of EMT, cell adhesion, migration, and invasion. Mechanistically, WFA inhibited TGFβ1-induced activation of Smad2/3-signaling and TNFα-dependent activation of NF-κB signaling in A549 and H1299 cells. In summary, our study provides additional evidence on the role of WFA in regulating EMT in human NSCLC cells and further demonstrates the therapeutic potential of WFA as an anti-metastatic agent for clinical application in NSCLC.

## Results

### WFA inhibits the growth and proliferation of NSCLC cells

**Figure 1:**
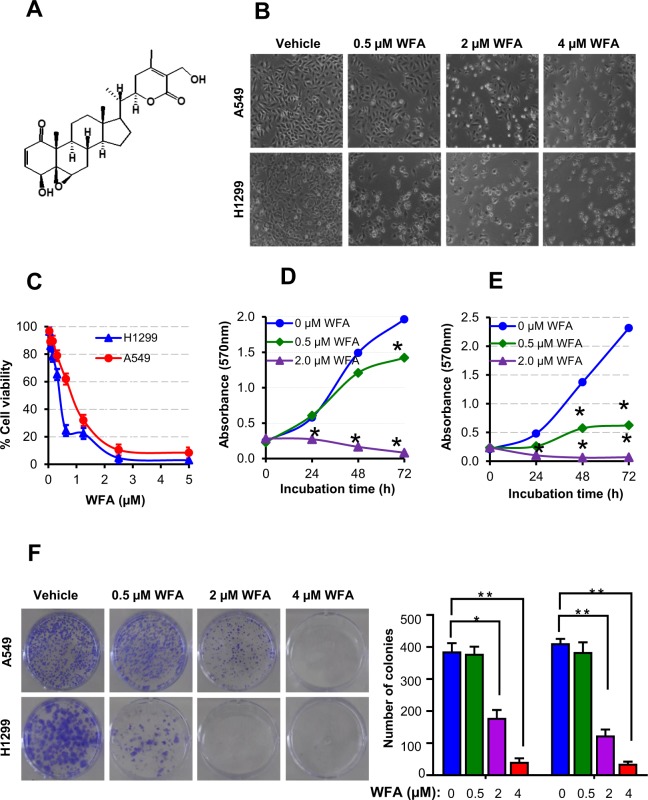
WFA suppressed the growth and proliferation of A549 and H1299 cells. (**A**) Chemical structure of WFA. (**B**) Phase-contrast images (4X magnification) showing the cellular morphology following incubation with WFA for 48 h. (**C**) The dose-dependent antiproliferative activity of WFA against A549 and H1299 cells after 72 h incubation. Time- and concentration-dependent inhibitory effects of WFA on the growth and proliferation of A549 (**D**) and (**E**) H1299 cells. (**F**) Representative images of colony formation assay depicting a dose-dependent inhibitory effect of WFA on A549 and H1299 cells. Cell viability was measured using MTT assay while the number of colonies formed was counted using a microscope and data are presented as mean ± SD of 3 technical replicates. *p < 0.05, **p < 0.01.

Figure 1 presents the data on the cytotoxicity of WFA against A549 and H1299 cells. Figure 1A shows the chemical structure of WFA depicting the overall arrangement of atoms into a steroid framework and 6-carbon lactone ring. Figure 1 panels B–F show the dose- and time-dependent cytotoxicity of WFA on A549 and H1299 cells. Phase contrast images in Fig. 1B depict changes in cellular morphology following incubation with WFA (0–4 µM) for 48 h. Clearly, the incubation of either cell lines with ≥2 µM WFA resulted in significant changes in cellular morphology indicative of decreased cell viability and/or increased cell death. On the other hand, incubation of cells with 0.5 µM WFA caused greater cytotoxicity to H1299 than A549 cells. In Fig. 1C–E, graphs show the quantitative cytotoxic effects of WFA on A549 and H1299 cells. Here, vehicle-treated cells were taken as 100% viable and used to calculate the relative cell viability of cells incubated in media containing various concentrations of WFA. In Fig. 1C, there was a dose-dependent decrease in the viability of both cell lines after incubation with WFA for 72 h. Similar to the data presented in Fig. 1B, the graphs in Fig. 1C also indicate that concentrations of WFA ≥ 2 µM resulted in up to 80% decrease in cell viability. Graphs in Fig. 1D,E show dose- and time-dependent inhibition in cell proliferation of both A549 and H1299 cells. Interestingly, the highly metastatic H1299 cells were more sensitive to WFA than the moderately metastatic A549 cells.

Next, to determine the effects of WFA on the replicative ability of cells, the colony formation assay was performed. In Fig. 1F, representative images show a dose-dependent decrease in the number of colonies formed by A549 and H1299 cells following incubation with WFA (additional images are presented in Supplementary Figs S1 and S2). Taken together, data presented demonstrate that WFA displays remarkable cytotoxicity against A549 and H1299 NSCLC cells *in vitro* but with greater potency against H1299 than A549 cells.

### WFA inhibits cell adhesion, migration, and invasion of NSCLC cells

**Figure 2:**
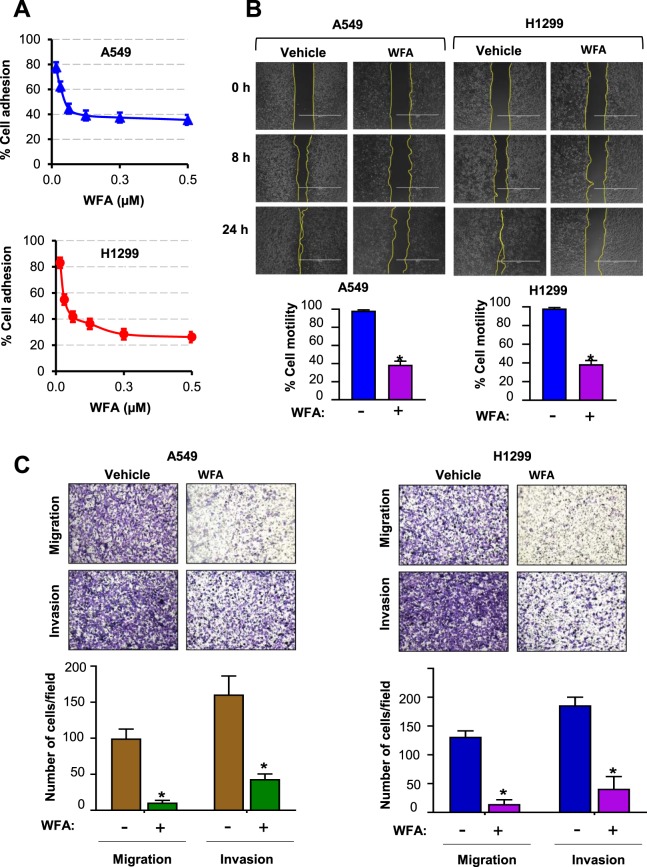
WFA inhibited cell adhesion, motility, migration, and invasion of A549 and H1299 cells. (**A**) Cell adhesion assay depicting the dose-dependent inhibition of the adhesion of A549 and H1299 cells. (**B**) Wound healing assay showing the inhibitory effect of WFA on the motility of A549 and H1299 cells. (**C**) Representative images showing the effect of WFA on transwell migration and invasion of A549 and H1299 cells following incubation with WFA. Data are mean ± SD, *p < 0.05.

Increased migratory and invasive behaviors of tumor cells are known to be indicative of a higher metastatic potential in NSCLC and other solid tumors. Therefore, to test whether WFA inhibits the metastatic potential of A549 and H1299 cells, we conducted the cell adhesion, migration, and invasion assays. Here, unlike in the cytotoxicity experiments in Fig. 1, cells were incubated with ≤0.5 µM WFA for ≤4 h to minimize cell death. In Fig. 2A, the results of the cell adhesion assay show the effects of WFA on the attachment of cells on to extracellular matrices. The viability of vehicle-treated cells (as measured by MTT assay) was taken as 100% cell adhesion and then used to determine the relative cell adhesion of the cells incubated in media containing indicated concentrations of WFA. The graphs show a dose-dependent inhibition of cell adhesion with up to 60% and 70% inhibition of the adhesion of A549 and H1299 cells, respectively at the highest concentration (0.5 µM) of WFA tested.

In Fig. 2B, pre-incubation of cells with 0.5 µM WFA significantly inhibited the motility of both A549 and H1299 cells. In the vehicle-treated groups, there was >95% coverage of the wound areas by cells within 24 h indicating higher cell motility. However, in the presence of WFA, only 20–40% of the wound areas were covered by cells indicating a significant (*p < 0.05) suppression of cell motility. Additionally, Fig. 2C shows the results of the transwell migration and invasion assays. Here, representative images of migrated and invaded cells as well as the number of cells (mean ± SD) counted in five random fields are shown. As shown, pretreatment of cells with 0.5 µM WFA prior to initiation of migration resulted in a statistically-significant (*p < 0.05) decrease in the number of migrated and invaded cells. The suppression of migration and invasion was greater in H1299 than A549 cells.

### WFA inhibited experimental EMT induction

**Figure 3:**
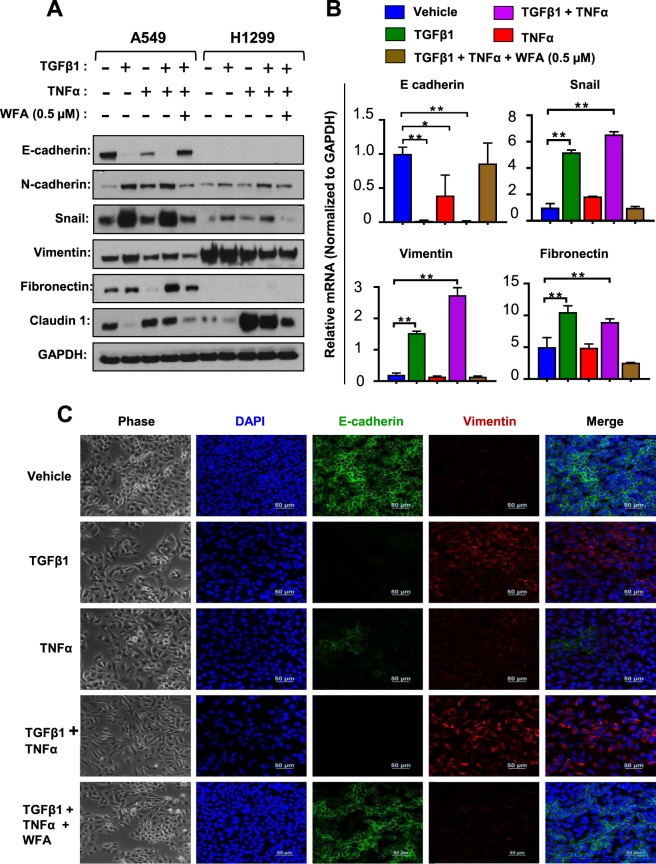
WFA inhibited experimental EMT induction in A549 and H1299 cells. Cells were left untreated or pretreated with 0.5 µM WFA for 12 h and then stimulated with TGFβ1 or TNFα alone and in combination in serum-free media. EMT induction was assessed by western blot analysis, qRT-PCR, and immunofluorescence staining. (**A**) Western blot analysis depicting the expression levels of the epithelial marker E-cadherin, and the EMT-related proteins N-cadherin, Snail, vimentin, and fibronectin (**B**) qRT-PCR depicting the relative mRNA expression. Fold change was calculated using the ^ΔΔ^Cт method and GAPDH was used as the normalizing gene. (**C**) Phase-contrast images (4x magnification) and confocal images showing changes in cellular morphology and the expression of E-cadherin and vimentin in A549 cells following incubation with a combination of TGFβ1 and TNFα in the presence or absence of 0.5 µM WFA. (*p < 0.05).

The loss of the epithelial marker E-cadherin and an increase in the mesenchymal protein N-cadherin, a phenomenon known as the cadherin switch, is the hallmark of EMT induction^13^. In previous studies, TGFβ1 and TNFα, either alone or in combination, have been used to experimentally induce EMT in various epithelial tumor cell types including NSCLC cells^38–40^. In the present study, we assessed whether WFA could inhibit TGFβ1 and TNFα-induced EMT in A549 and H1299 cells. First, EMT induction was achieved by culturing cells in serum-free media containing TGFβ1 (5 ng/mL) and TNFα (25 ng/mL) alone or in combination. In Fig. 3A, western blot analysis of whole cell lysates at 48 h following EMT induction shows that A549 but not H1299 cells expressed E-cadherin. Incubation of A549 cells with TGFβ1 and TNFα alone or in combination resulted in a significant decrease in the expression of E-cadherin in A549 cells while upregulating the expression of N-cadherin (Fig. 3A). The qRT-PCR data in Fig. 3B show that the repression of E-cadherin was also associated with decreased levels of mRNA normalized to GAPDH. On the other hand, there was a significant increase in the expression of the EMT-related proteins vimentin, Snail, fibronectin, and claudin-1 (Fig. 3A,B). Paradoxically, only TGFβ1 or its combination with TNFα increased the expression of Snail and vimentin expression. Additionally, Fig. 3A shows that the basal levels of vimentin were greater in H1299 than in A549 cells and that the effects of TGFβ1 and TNFα on the expression of vimentin in H1299 cells were either minimal or even slightly antagonistic.

When cells were pre-incubated with 0.5 μM WFA prior to TGFβ1 and TNFα stimulation, the observed EMT induction was suppressed. In Fig. 3A, western blot analysis shows that cells incubated with WFA resisted the loss of E-cadherin or the upregulation of vimentin, Snail, fibronectin, and claudin-1. The relative protein band intensities of the gels presented in Fig. 3A normalized to GAPDH are presented in the Supplementary Tables S1a and S1b for A549 and H1299 cells, respectively. The mean CT values indicating the relative expression of the normalizing genes (GAPDH and beta-actin) related to Fig. 3B are presented in the Supplementary Table S1c.

To validate the western blot and qRT-PCR data, we performed immunofluorescence staining for E-cadherin and vimentin in A549 cells. In Fig. 3C, phase contrast and confocal images are shown depicting the changes in cell morphology indicative of loss of cell to cell contact after incubation with TGFβ1 and TNFα. In agreement with the data presented in Fig. 3A,B, the images show that TGFβ1 and TNFα caused a downregulation of E-cadherin and an upregulation of vimentin, confirming the induction of EMT. As was observed in the western blot and qRT-PCR data, the confocal images also revealed that the combination of TGFβ1 and TNFα synergistically inhibited E-cadherin expression. More importantly, cells pre-incubated with WFA did not show significant changes in cell morphology, the loss of E-cadherin or increase in vimentin expression.

**Figure 4:**
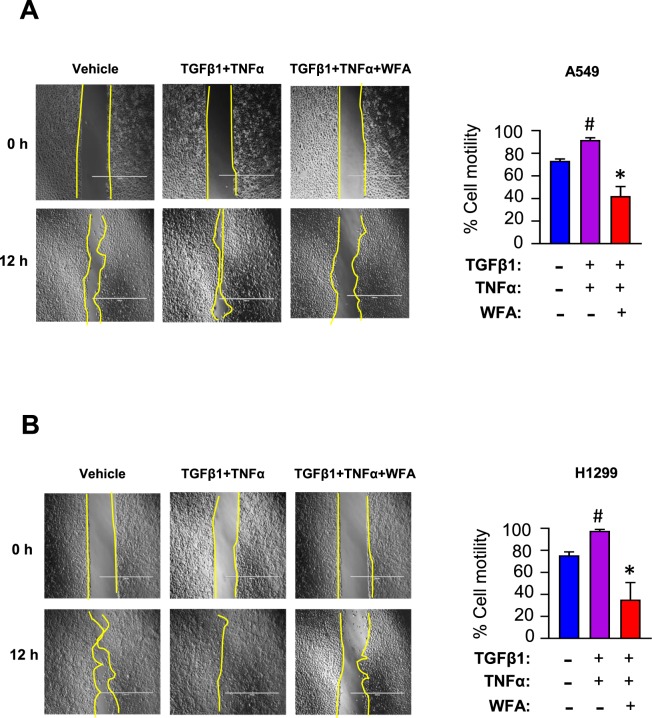
WFA suppressed TGFβ1/TNFα-induced motility of A549 and H1299 cells. Wound healing assay was used to measure the motility of the cells in the presence of 0.5 µM WFA and a combination of TGFβ1 (5 ng/mL) and TNFα (25 ng/mL). The figure shows that the cytokines TGFβ1 and TNFα significantly enhanced the motility of A549 (**A**), and H1299 **(B)** cells. However, in the presence of WFA, TGFβ1/TNFα-induced motility was inhibited. Data are presented as percent migration (mean ± SD) by considering wound area at time 0 h as 100%. (*p < 0.05).

To determine the effect of WFA on TGFβ1- and TNFα-induced cell motility, the wound healing assay was conducted with cells cultured in serum-free media in the presence of TGFβ1 and TNFα. In Fig. 4, TGFβ1 and TNFα stimulated >90% migration within 12 h (#p < 0.05 vs vehicle treatment). However, in the presence of 0.5 µM WFA, the cytokine-induced cell motility was significantly (*p < 0.05 vs TGFβ1/TNFα group) suppressed in both cell lines.

### WFA inhibited TGFβ1-dependent Smad2/3 signaling

**Figure 5:**
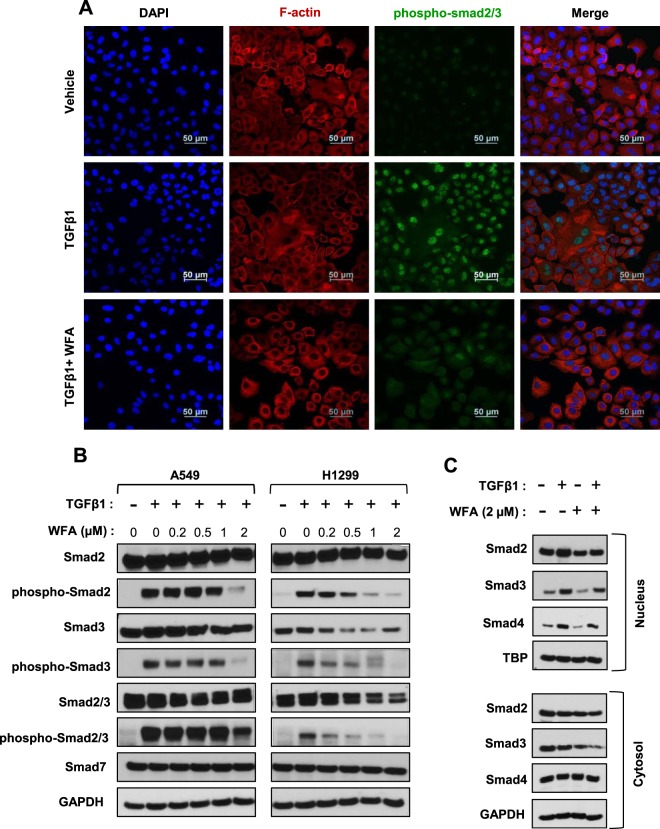
WFA inhibited TGFβ1-dependent activation of Smad2/3 in A549 and H1299 cells (**A**) Confocal images of A549 NSCLC cells depicting the inhibitory effect of WFA (1.0 µM) on TGFβ1 (5 ng/mL)-induced Smad2/3 phosphorylation in A549 cells. (**B**) Western blot analysis of whole cell lysates depicting the dose-dependent inhibitory effect of WFA on the Smad2/3 signaling pathway (**C)** Western blot analysis of cytosolic and nuclear fractions from A549 cells showed decreased Smad2/3 phosphorylation and nuclear translocation in the presence of WFA. GAPDH was used as the total protein loading control.

It has been shown that TGFβ1 regulates EMT induction mainly through the activation of Smad2/3-dependent signaling pathways^41^. Therefore, to assess if WFA inhibited TGFβ-induced Smad2/3 activation, cells were incubated with WFA at indicated concentrations and then stimulated with TGFβ1. The phosphorylation of Smad2/3 was assessed by immunofluorescence staining and western blot analysis. Figure 5A shows confocal images of A549 cells stimulated with TGFβ1 for 30 minutes and then probed for phospho-Smad2/3 (green), F-actin (red) and nuclei (blue). Additional images presented in the supplementary information (Fig. S3) show that cells stimulated with TGFβ1 for 1 h had increased levels of phospho-Smad2/3 and its nuclear localization when compared to vehicle-treated cells. However, pre-incubation of cells with WFA prior to stimulation with TGFβ1 suppressed the phosphorylation and nuclear translocation of Smad2/3 (Fig. 5A and Supplementary Fig. S3). We performed western blot analysis on whole cell lysates to determine the effect of WFA on Smad2/3 activation. As shown in Fig. 5B, TGFβ1 stimulated the phosphorylation of Smad2 and Smad3 in A549 and H1299 as compared to serum-starved cells (control group). In both cell lines, WFA dose-dependently suppressed Smad2 and Smad3 phosphorylation. Moreover, it appears that WFA also decreased the levels of total Smad3 in a dose-dependent manner in A549 and H1299 cells. Based on relative band intensities presented in Supplementary Tables S2a–d, incubation of cells with 2 µM WFA resulted in up to 80% inhibition of the phosphorylation of Smad2 and Smad 3. Interestingly, WFA treatment did not affect the levels of Smad7, the inhibitor of Smad2/3 activation. Analysis of the nuclear and cytosolic fractions of A549 cells indicated that TGFβ1 stimulation increased the nuclear translocation of Smad2, Smad3 and Smad4 (Fig. 5C). However, 2 µM WFA suppressed both the basal and induced nuclear translocation of Smad2, Smad3 and Smad4. Images of the full-length western blot gels of the whole cell lysates, nuclear, and cytoplasmic fractions are presented in Supplementary Figs S4 and S5.

### WFA inhibited TNFα-induced NF-κB signaling

**Figure 6:**
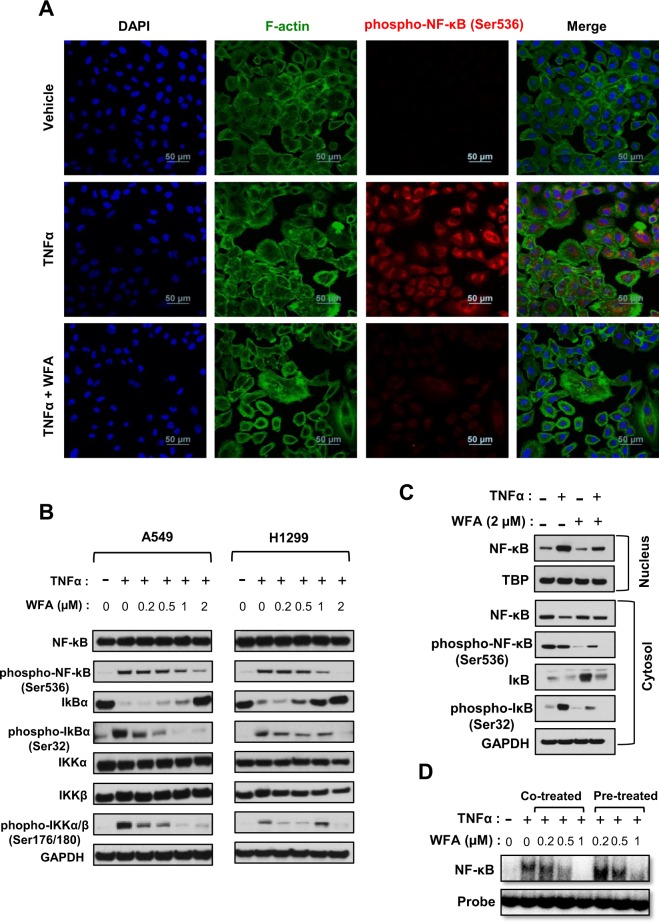
Inhibition of TNFα-induced NFκB activation in human NSCLC cells. (**A**) Confocal images (40x magnification) showing the levels of p-NF-κB. (**B**) Western blot analysis depicting levels of p-NFκB, total NF-kB, p-IκB, and total IκB in whole cell lysates following stimulation with TNFα (25 ng/mL (**C**) Western blot analysis of nuclear and cytosolic cell extracts depicting levels of NFκB, p-NFκB, IκBα, and p-IκBα following stimulation with TNFα. (**D**) Electrophoretic mobility shift assay, indicating the inhibition of NF-κB binding to DNA. Cells were incubated with WFA for 4 h and then stimulated with TNFα (25 ng/mL). Band intensity represents the extent of DNA binding.

In tumor cells, TNFα stimulates NF-κB signaling pathways to regulate motility, migration, invasion, and chemoresistance^38^. In the present study, we investigated whether WFA inhibited TNFα-induced activation of NF-κB in H1299 and A549 cells. As shown in Fig. 6, we observed an increase in the phosphorylation and nuclear translocation of NF-κB following TNFα stimulation. Immunofluorescence staining in Fig. 6A shows that cells stimulated with TNFα had higher levels of phospho-NF-κB than vehicle treatment. However, pre-incubation of cells with WFA significantly decreased the levels of phospho-NF-κB. In Fig. 6B,C, western blot analysis of the whole cell and cytosolic lysates indicates that TNFα alone increased the levels of phospho-IκBα and phosphor-NFκB (p-NF-κB). The phosphorylation of IκB resulted in increased degradation of IκB and nuclear translocation of NF-κB (Fig. 6C). WFA suppressed the TNFα-dependent phosphorylation of NF-κB and IκBα, and the degradation of IκBα and nuclear translocation of NF-κB (P65). Interestingly, the total levels of NF-κB were not significantly affected by WFA indicating that WFA inhibited NF-κB signaling by regulation of phosphorylation. By measuring the levels of total and phosphorylated IKKα/β, we found that WFA inhibited NF-κB (P65) by regulating the activity of IKKα/β. The full-length gels and relative band intensities related to Fig. 6 are presented in the Supplementary Fig. S6 and Supplementary Table S3a–d, respectively. Furthermore, we also investigated the effect of WFA on the transcriptional activity of NF-κB (P65) using the electrophoretic mobility shift assay (EMSA). Our data on EMSA in Fig. 6D show that WFA decreased the binding of NF-κB (P65) on to DNA in both A549 and H1299 cells. Interestingly, N-acetyl cysteine (NAC) abrogated the effects of WFA on NF-κB signaling as shown in the Supplementary Fig. S7. By comparison, the potency of WFA on NF-κB signaling inhibition was greater in H1299 than A549 cells. Overall, our results demonstrate that WFA remarkably inhibited TNFα-induced degradation of IκB, the phosphorylation and nuclear translocation of NF-κB without affecting total NF-κB levels in both A549 and H1299 cells.

## Discussion

Despite the recent advances in the treatment of NSCLC such as targeted therapies and immunotherapies, invasion and metastasis remain a major therapeutic challenge^5^. Recently, it has become clear that EMT plays a crucial role in the metastasis of NSCLC tumors and therefore has become an attractive drug target^12^. Indeed it has been shown that EMT induction causes tumor cells to become more migratory, invasive, and metastatic^14^ Additionally, EMT stimulates tumor cells to acquire stem cell-like properties and increases their resistance to standard chemotherapeutic drugs^42^. Therefore, the importance of EMT during metastasis and in drug resistance of tumor cells has prompted the search for compounds that can inhibit EMT in tumor cells as potential drugs for metastatic NSCLC^43^. Based on this hypothesis, we and others^27,29,34^ have examined the effects of WFA on experimentally induced EMT to demonstrate its clinical potential as an anti-metastatic agent.

WFA is a member of the withanolides, a large group of naturally occurring 28-carbon-containing compounds^44^. The chemical structure of withanolides is composed of a steroidal framework attached to a lactone ring, and are sometimes referred to as ‘steroidal-lactones’^45^. In the WFA structure (Fig. 1A), there are five functional groups including; an unsaturated ketone ring, 2 hydroxyl groups, an epoxide ring, a 6-carbon lactone ring, and an unsaturated carbonyl group^46,47^. These five functional groups enable WFA to interact with several molecular targets and account for its diverse biological activities^17^. Today, multiple lines of experimental evidence from *in vitro* and *in vivo* studies show that WFA is a highly efficacious anti-tumor molecule that displays cytotoxic activities against multiple cancer cells^20,25,48^. Notably, WFA also inhibits *in vitro* tumor cell migration, invasion, and metastasis^17^ which have stimulated the widespread interest in investigating its anti-metastatic properties. Unlike common cytotoxic chemotherapeutic drugs that have been shown to induce EMT^49^, WFA, on the other hand, has been reported to suppress EMT^27,29,34,37^ and therefore has the capability to prevent metastasis in NSCLC and other cancers.

Earlier studies have shown that sub-cytotoxic doses of WFA inhibit the induction of EMT in breast^27,34,35^, ovarian^29^, lung^36^, and melanoma^37^ cancer cells. In fact, detailed molecular evidence indicates that WFA binds to and causes the degradation of the EMT-related intermediate filament protein, vimentin^27,50^. Similarly, our study highlights the role of WFA in regulating EMT and metastasis in NSCLC cells. As reported elsewhere^20^, our findings show remarkable cytotoxicity of WFA on the anchorage-dependent growth and proliferation of NSCLC cells. The highly invasive H1299 cells^51^ were found to be more sensitive to WFA than the moderately invasive A549 cells. This finding suggests and strengthens our hypothesis that WFA is likely more potent against the highly metastatic tumors which are incidentally found in the majority of NSCLC patients.

During EMT, tumor cells undergo changes that results in loss of cell to cell contacts, become more invasive and resistant to cytotoxic drugs^52^. At the molecular level, it has been shown that the loss of the epithelial marker E-cadherin coupled with an increase in mesenchymal markers such as vimentin and N-cadherin are the hallmarks of EMT^12^. Furthermore, EMT pathways are regulated by various extracellular signals in the tumor microenvironment such as TGFβ1 and TNFα^52,53^. Therefore, experimentally, EMT induction in tumor cells has been modeled by culturing cells in serum-free media containing TGFβ1 and/or TNFα^14^. In agreement with previous studies^54^, our findings show a synergistic interaction between TGFβ1 and TNFα on EMT induction as indicated by E-cadherin repression. Interestingly, pre-incubation of cells with WFA prior to EMT induction prevented the loss of E-cadherin or the upregulation of N-cadherin. Together with previous data^27,34,55^, our study also demonstrates that WFA is a powerful suppressor of EMT and further highlights the potential anti-metastatic effects of WFA.

The molecular mechanisms underlying EMT are complex but the transcription factor Snail has been shown to be a key regulator of EMT induction in NSCLC cells^53^. Specifically, Snail binds to DNA and causes the repression of E-cadherin expression while upregulating EMT-related proteins such as vimentin, N-cadherin, and fibronectin^14,42,53^. On the other hand, Snail expression and function are regulated by extracellular signals such as TGFβ1^12,14^ and TNFα^54^ via Smad and NF-κB signaling mechanisms, respectively. Moreover, it has been shown that simultaneous activation of these two pathways synergistically increases the expression of Snail in A549 cells^41,54,56^. Indeed, our data demonstrated that the combination of TGFβ1 and TNFα synergistically increased the expression of Snail which explains the observed loss of E-cadherin and upregulation of N-cadherin. However, in the presence of WFA, the cytokine-induced Snail expression was inhibited and thus EMT induction was suppressed. Overall, these findings suggest that the observed inhibition of EMT induction by WFA occurs at least in part due to the regulation of Snail expression by inhibiting Smad and/or NF-κB signaling pathways.

To further understand the potential mechanisms underlying the inhibitory effects of WFA on TGFβ1/TNFα-dependent EMT induction, we explored Smad and NF-κB signaling pathways. Smad2 and Smad3 belong to a family of transcription factors that exist as a Smad2/3 dimer in the cytoplasm in the inactive (dephosphorylated) form. However, following TGFβ1 stimulation, these are phosphorylated and then bind to another cytosolic protein called Smad4 and co-translocate to the nucleus where they stimulate the expression of Snail^53^. Therefore, to determine whether WFA inhibits the activation of Smad signaling, we measured the levels of total and phosphorylated Smad2, Smad3, and Smad4 in the whole cell, cytoplasmic, and nuclear fractions. Although WFA has already been shown to inhibit Smad2/3 phosphorylation^55^, our study provides additional evidence on the multiple effects of WFA on Smad signaling. WFA inhibited the phosphorylation of both Smad2 and Smad3, and thus prevented their nuclear translocation. Additionally, it appears that WFA also targeted Smad3 and caused its degradation.

Several published studies have shown that WFA is a potent anti-inflammatory compound and has been examined for its effects on NF-κB signaling mechanisms^17,48,57,58^. In tumor cells, TNFα activates the NF-κB signaling to stimulate EMT induction, cell survival, proliferation, migration, and chemoresistance^38,56^. Mechanistically, the activation of NFκB involves two major events; (1) the IKKα/β dependent phosphorylation and subsequent degradation of IκBα, the inhibitor of NF-κB (P65), and (2) the phosphorylation and nuclear translocation of NF-κB (P65). Data from previous studies^57^ conducted on fibrosarcoma and human embryonic kidney cells indicated that WFA regulates NF-κB signaling by inhibiting the kinase activity of IKKα/β. This resulted in decreased phosphorylation and degradation of IκBα, the phosphorylation and nuclear translocation of NF-κB (P65). Similarly, we also observed a dose-dependent inhibition of TNFα-induced activation of NF-κB signaling in both A549 and H1299 cells. The phosphorylation and degradation of IκBα was inhibited concomitant with decreased NF-κB phosphorylation, nuclear translocation, and binding to DNA. Our findings provide additional evidence on the inhibitory effect of WFA on NFκB signaling mechanisms via the interference with IKKα/β kinase activity.

In conclusion, our study demonstrates the inhibitory activity of WFA on EMT induction, adhesion, migration, and invasion of NSCLC cells. Our data provide evidence to show that WFA regulates TGFβ1 and TNFα-induced EMT via the regulation of Smad and NFκB signaling pathways. Together, the findings presented here suggest that WFA is a promising antitumor agent that has therapeutic potential in the treatment of metastasis in NSCLC and other cancers. Additionally, our observation that WFA was more potent against the highly-invasive H1299 cells than the moderately-invasive A549 cell line further strengthens this hypothesis.

## Materials and Methods

### Reagents, chemicals, and supplies

WFA (>96% pure) was generously provided as a gift sample by 3P Biotechnologies Inc., (Louisville, KY, USA). A stock solution of WFA (5 mM) was prepared in dimethyl sulfoxide (DMSO) and stored in aliquots at −20 °C until use. MTT (3-[4,5-dimethylthiazol-2-yl]−2,5-diphenyltetrazolium bromide) was purchased from Alfa Aesar (Ward Hill, MA, USA). RIPA cell lysis buffer, BCA protein assay kit, PVDF membranes, phosphate–buffered- saline (PBS), ECL chemiluminescence reagent, and Bolt 4–12% Bis-Tris phosphate gels were purchased from ThermoFisher (Rockford, IL, USA). Dulbecco’s Modified Eagle’s Medium (DMEM), penicillin and streptomycin were purchased from Life Technologies (Gibco, Grand Islands, NY, USA). Fetal bovine serum (FBS) was purchased from VWR, Seradigm Life Science (Radnor, PA, USA). The human recombinant TGFβ-1 (#8915) and TNFα (#8902) were purchased from Cell Signaling Technology (Danvers, MA, USA) and prepared as 50 µg/mL stock solutions in sterile 20 mM citrate, pH 3.0 and sterile PBS, respectively. The stock solution was stored in aliquots at −80 °C and diluted in serum-free DMEM before use.

### Antibodies

Primary antibodies against Smad2/3, phospho-Smad2/3, Smad2, Smad3, phospho-Smad2, phospho-Smad3, Smad4, Smad7, Snail, Slug, E-cadherin, ZEB1, N-cadherin, Vimentin, NF-κB, pNFκB, IκB, phospho-IκB, IKKα, IKKβ, phospho-IKKα/β, and were purchased from Cell Signaling Technology (Danvers, MA). The primary antibodies against β-actin were purchased from Sigma Chemical Co. (St. Louis, MO). A detailed list of all antibodies used is presented in Supplementary Table S4.

### Cells and culture conditions

The human NSCLC cell lines, A549, and H1299 were maintained in DMEM media supplemented with 10% (*v/v*) fetal bovine serum (FBS) and 1% antibiotics (100 U/mL penicillin and 100 µg/mL streptomycin), at 37 °C with 5% CO_2_ in a humidified incubator. Culture media was replaced every 48 h and the cells were passaged at ∼80% confluence, for less than 20 cycles.

### Cell viability assay

Cell viability was determined by MTT assay as described previously^59,60^. Briefly, A549 and H1299 cells were seeded in 96-well plates (3 × 10^3^ cells/well in 100 μL of DMEM) and pre-incubated for 24 h before treatments. Then, DMEM was replaced with fresh media containing various concentrations [0–5 µM] of WFA and incubated for an additional 24, 48, and 72 h. After incubation for the desired time points, the media containing WFA was replaced with media containing 0.5 mg/mL MTT reagent and incubated for 3 h. The purple formazan crystals formed were solubilized using 200 µL of DMSO and the absorbance of the resulting solution was measured at 570 nm. The optical density (OD) values of the vehicle-treated cells were taken as 100% cell viability and used to calculate the relative viability of cells incubated with WFA. Data were expressed as mean ± SD of three separate experiments.

### Cell adhesion assay

Cells were serum starved for 12 h and then incubated with WFA in the presence or absence of TGFβ1 or TNFα in serum-free media for 1 h. The cells were then trypsinized and seeded in Matrigel (50 μg/mL) pre-coated 96-well plates (5 × 10^4^ cells/well) and incubated for 2 h to allow attachment. The non-adherent cells were washed off using pre-warmed sterile PBS, and then fresh complete DMEM (10% FBS) was added to each well. The cells were incubated for an additional 4 h, and then cell viability was measured using MTT assay. The viability of cells in the vehicle-treated group was considered as 100% cell adhesion and used to calculate the relative adhesion of the cells incubated with WFA.

### Colony formation assay

Cells were incubated with or without WFA for 24 h, trypsinized and then seeded in 6-well plates at 500 cells/well density. DMEM was replaced every 48 h for a total of 10 d. The colonies formed were fixed using methanol/acetic acid solution (3:1) and then stained with 0.5% crystal violet in methanol. The number of colonies formed was recorded using a high-resolution digital camera and counted using a microscope in five random fields. The number of colonies from each treatment group is presented as mean ± SD of three technical replicates.

### Wound healing assay

The wound healing assay was performed using the 2-well inserts from ibidi® (Munich, Germany) placed in 6-well plates. Briefly, cells were suspended in culture media (100 cells/μL) and 100 μL of the cell suspension was added to each of the 2 wells in the cell inserts. The cells were incubated for 24 h to allow cell attachment and then cell-free gaps (wounds) were created by removing the culture inserts. Floating cells and cellular debris were washed off using pre-warmed sterile PBS, and then fresh media containing 0.5 µM WFA in the presence or absence of TGFβ1 and TNFα was added. The wound areas (cell-free areas) were monitored by light microscopy at 0, 12, and 24 h. At each of these time point, 3 microphotographs were taken and the wound areas for each photograph were quantitatively determined using Wimasis Image Analysis software (WimScratch, Cordoba, Spain).

### Transwell migration and invasion assays

The transwell migration and invasion assays were performed using uncoated and matrigel-coated 8 µm pore size transwell chambers (BD Bioscience, San Jose, CA), respectively. Cells were pretreated with WFA for 1 h then harvested by trypsinization and counted under the microscope. In the migration assay, 4 × 10^4^ cells were suspended in 200 μL of serum-free DMEM and seeded into each of the top compartments of the transwell chambers. Complete DMEM (10% FBS) was added to the bottom chamber to act as a chemoattractant to cause cell migration of cells from the top to the bottom compartments of the transwell chamber. Cell migration was allowed to occur for 24 h, then the migrated cells were fixed using 4% paraformaldehyde, permeabilized using 100% methanol, and stained using 0.2% toluidine blue. The number of migrated cells were counted in five random fields under a microscope. Data are presented as representative microphotographs and mean ± SD of the number of migrated cells. In the invasion experiment, the same procedure was performed except with 8 × 10^4^ cells/well and with Matrigel pre-coated transwell chambers.

### Western blot analysis

Cell lysates were prepared using RIPA cell lysis buffer at 4 °C. Total cellular protein concentrations were determined for each treatment group by BCA method and 20 µg protein samples were resolved by SDS-PAGE. Proteins separated according to molecular weights were transferred to PVDF membranes and probed for the expression of specific proteins (The list of antibodies used is presented in Supplementary Table S4). The levels of each protein of interest were determined by visualizing protein bands using ECL detection reagents (Thermo Scientific, Rockford, IL). The densities of each protein band relative to the internal loading control (GAPDH) were quantified using ImageJ software (NIH, Bethesda, MD).

### Electrophoretic mobility shift assay (EMSA)

The DNA binding characteristics of NF-κB were assessed using EMSA as previously described^60^. Briefly, cells were incubated with or without WFA (0–1 µM) for 12 h and then stimulated with 25 ng/mL TNFα in serum-free media for 30 min. Nuclear cell extracts were prepared and a total of 30 µg nuclear protein was incubated with 0.2 μg of ^32^P-end-labeled double-stranded oligo nucleotide (5′-AGT TGA GGG GAC TTT CCC AGG C-3′) containing the NF-κB binding motif (Promega, Madison, WI, USA) for 45 minutes as per the manufacturer’s instructions. The DNA–protein complexes formed were separated from free oligonucleotide by electrophoresis on a 6% native polyacrylamide gels and the DNA-NFκB complex radioactive bands were visualized by a Packard InstantImager (Downers Grove, IL).

### Immunofluorescence staining

Cells were seeded in 8-well chamber slides and cultured up to 60–70% confluence. After 24 h serum starvation, cells were incubated with TGFβ1 or TNFα in the presence or absence of WFA for 12 h. The cells were then fixed using 4% paraformaldehyde, blocked for 1 h in 1% BSA/ 0.25% Triton X-100 in PBS at room temperature. The cells were probed with specific primary antibodies at 4 °C overnight and then followed by specific fluorescent-tagged secondary monoclonal antibodies diluted in 1% BSA in PBS for 2 h at room temperature. F-actin was stained using green or red fluorescent phalloidin while the nuclei were stained blue using DAPI (ThermoFisher, Rockford, IL).

### qRT-PCR analysis

Total RNA was isolated from cells using Trizol reagent (Invitrogen, Carlsbad, CA). RNA samples (200 ng) were reverse transcribed and amplified using Power SYBR Green RNA to CT 1-Step Kit (Applied Biosystems). The relative mRNA expression levels of E-cadherin, vimentin, Snail, and fibronectin normalized to GAPDH or beta-actin were determined. The list of primers used is presented in the Supplementary Table S5 and the fold change for each sample was determined using the 2^−ΔΔCt^ relative quantification method^60^

### Statistical analysis

Where indicated, data are presented as means ± SD and statistical analysis was performed using Graph Pad Prism 7.0 software. Comparisons between experimental groups were made using the students’ t-test and p-values less than 0.05 were considered statistically significant. Quantification of western blot analysis presented in supplementary information is from a single set of experiment.

## Electronic supplementary material

Supplementary Information
