# Supplementary material for: Withaferin A inhibits Epithelial to Mesenchymal Transition in Non-Small Cell Lung Cancer Cells
Source: Sci Rep. 2018 Oct 24;8:15737. doi: 10.1038/s41598-018-34018-1 (PMC6200817; doi:10.1038/s41598-018-34018-1)
Supplement: Supplementary file 1 — Supplementary Information [file 41598_2018_34018_MOESM1_ESM.pdf]

Supplementary Materials

<u>Title</u>:

Withaferin A inhibits Epithelial to Mesenchymal Transition in Non-Small Cell Lung Cancer Cells

Author list and affiliation:

Al-Hassan Kyakulaga[1], Farrukh Aqil[2, 3], Radha Munagala[2, 3], and Ramesh C. Gupta[1,3,*]

[1]Department of Pharmacology and Toxicology, [2]Department of Medicine, [3]James Graham Brown Cancer Center, University of Louisville, Louisville, KY 40202

***Corresponding author:**

Ramesh C. Gupta, Delia Baxter II, Room 304E, University of Louisville, 580 S. Preston Street, Louisville, KY 40202. Email: rcgupta@louisville.edu; Tel: 502 852 3682, Fax: 502 852 3842

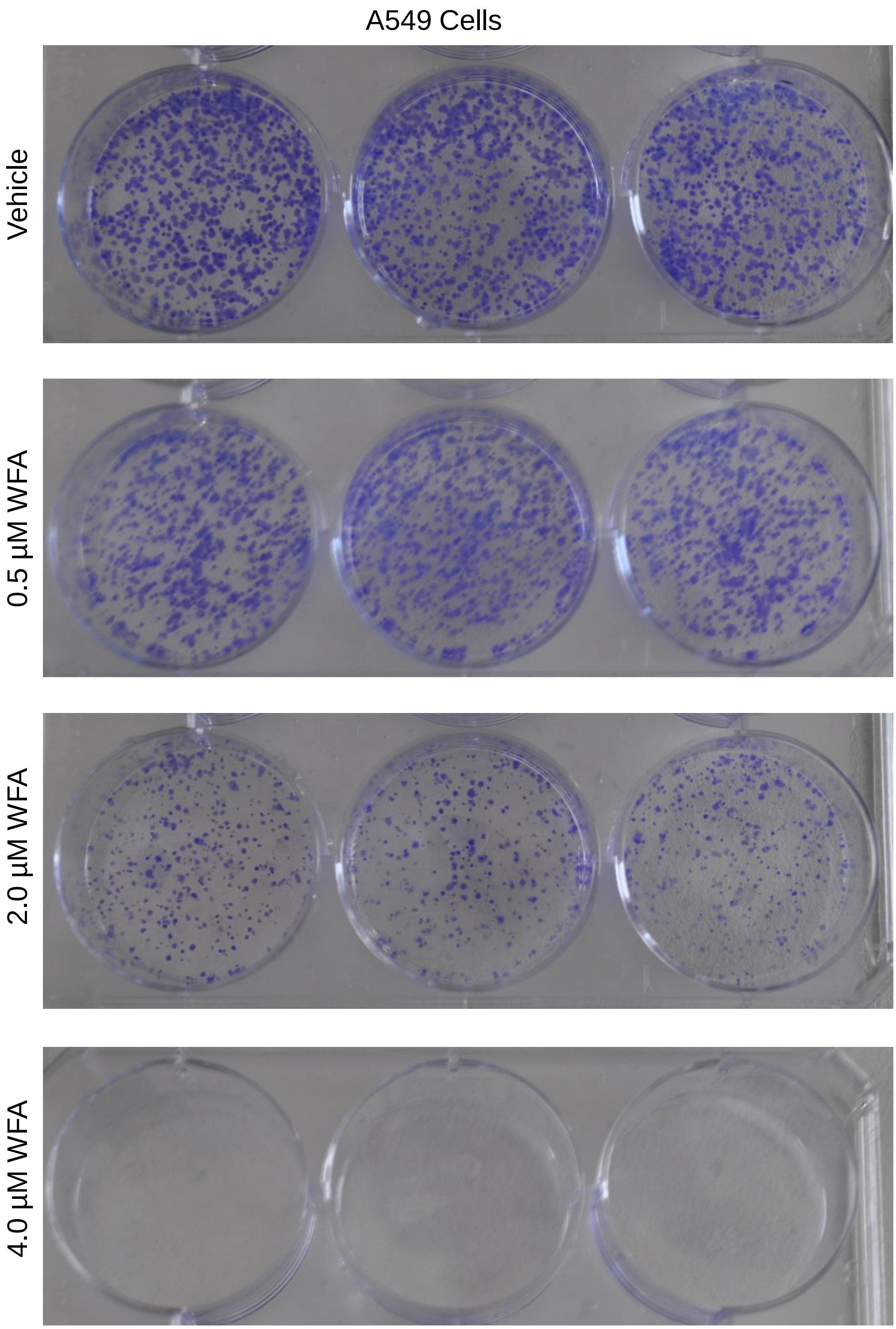

Supplementary Figure S1. Unedited images showing dose-dependent inhibition of colony formation in A549 cells related to Figure 1F. Cells were incubated with WFA (0-4 µM) in triplicates for 24 h and then incubated in fresh media for an additional 10 days. Colonies were stained 0.5% crystal violet.

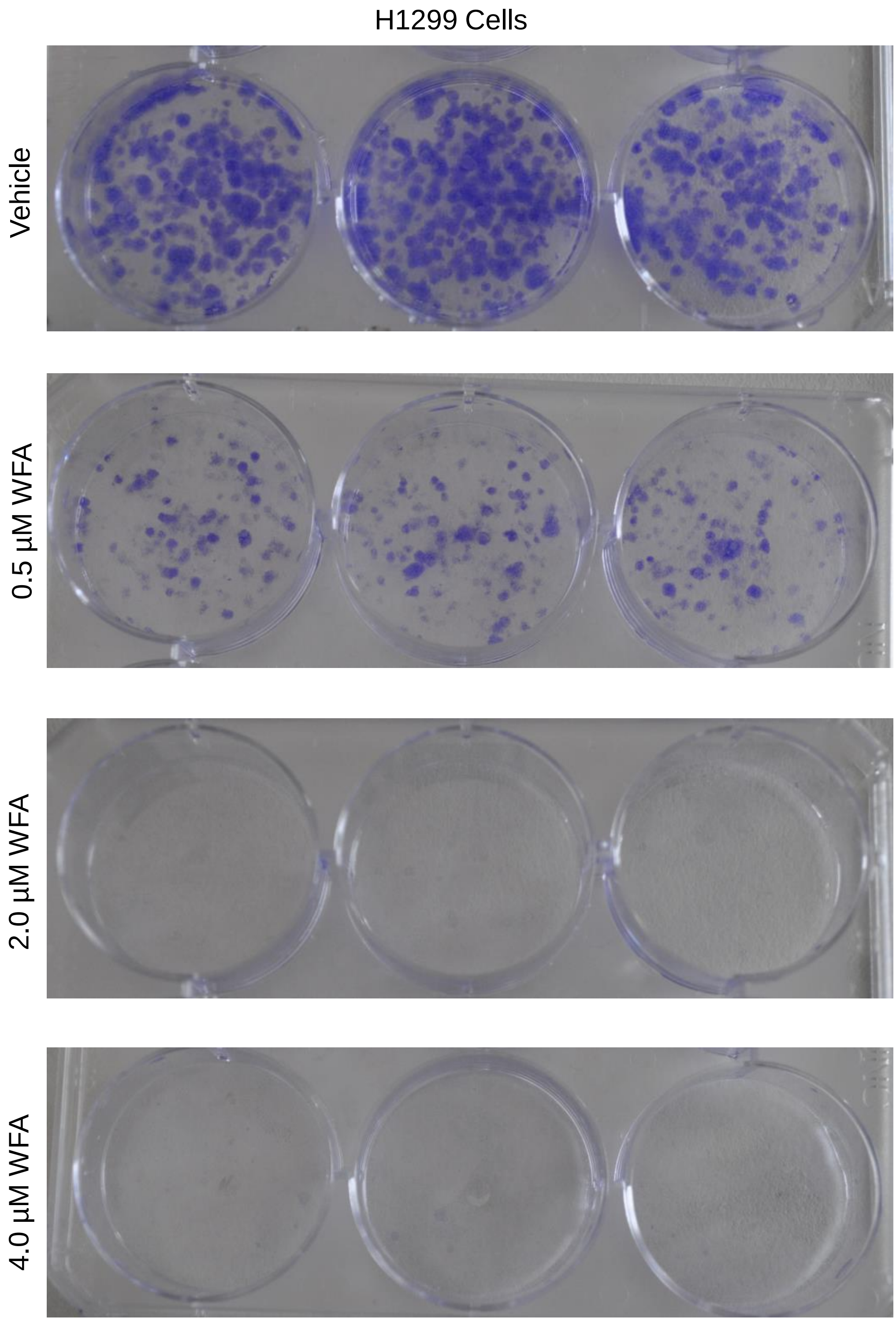

Supplementary Figure S2. Unedited images showing dose-dependent inhibition of colony formation in H1299 cells related to Figure 1F. Cells were incubated with WFA (0-4 µM) in triplicates for 24 h and then incubated in fresh media for an additional 10 days. Colonies were stained 0.5% crystal violet.

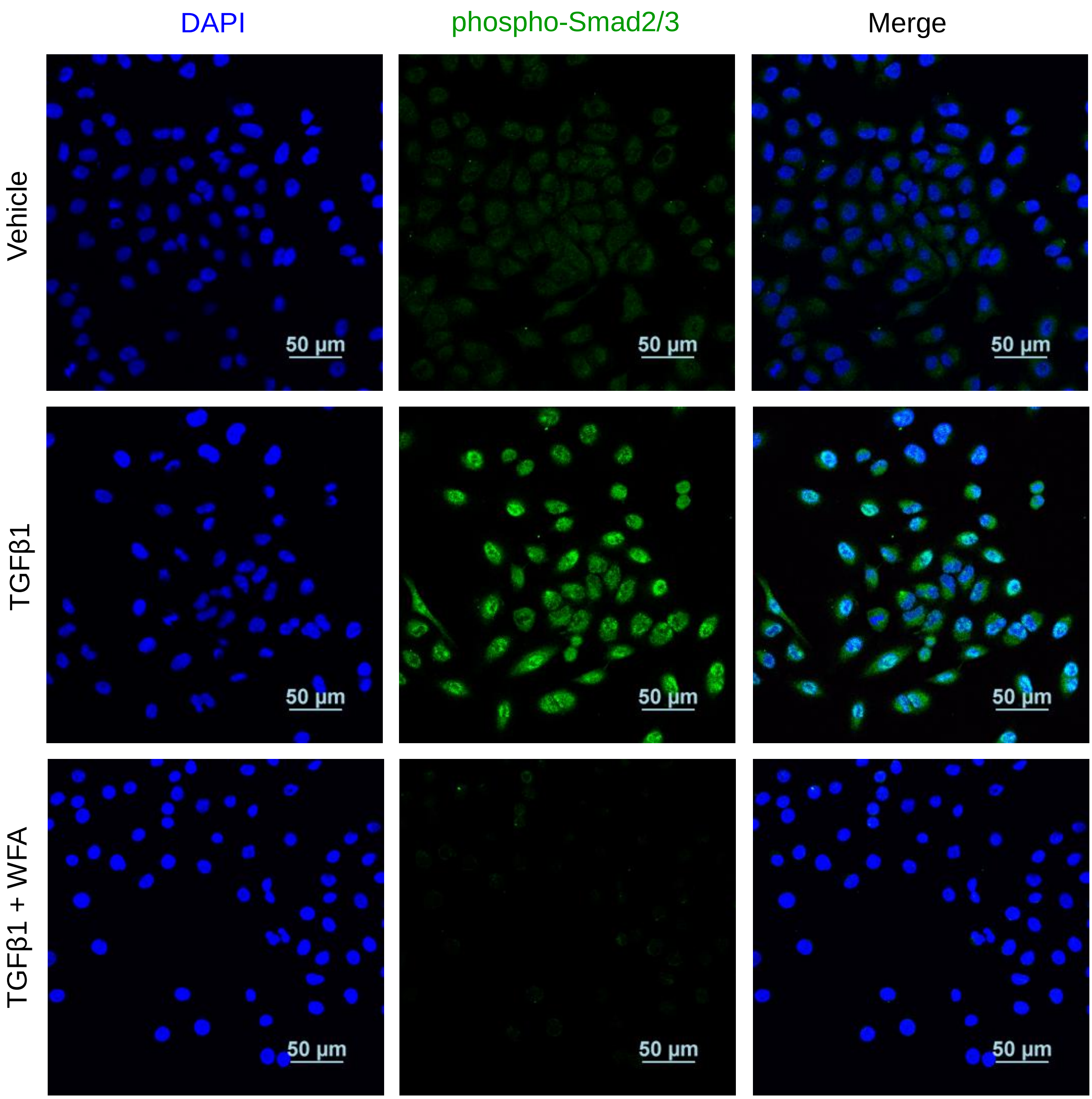

Supplementary Figure S3. Representative confocal images of A549 cells stimulated with TGFβ1 for 1 h depicting the phosphorylation and nuclear translocation of Smad2/3. The images show cells probed for phospho-Smad2/3 (green) nuclei (blue). Incubation of cells with WFA (0.5µM) decreased both the phosphorylation and nuclear translocation of Smad2/3 in A549 cells.

Whole cell lysates

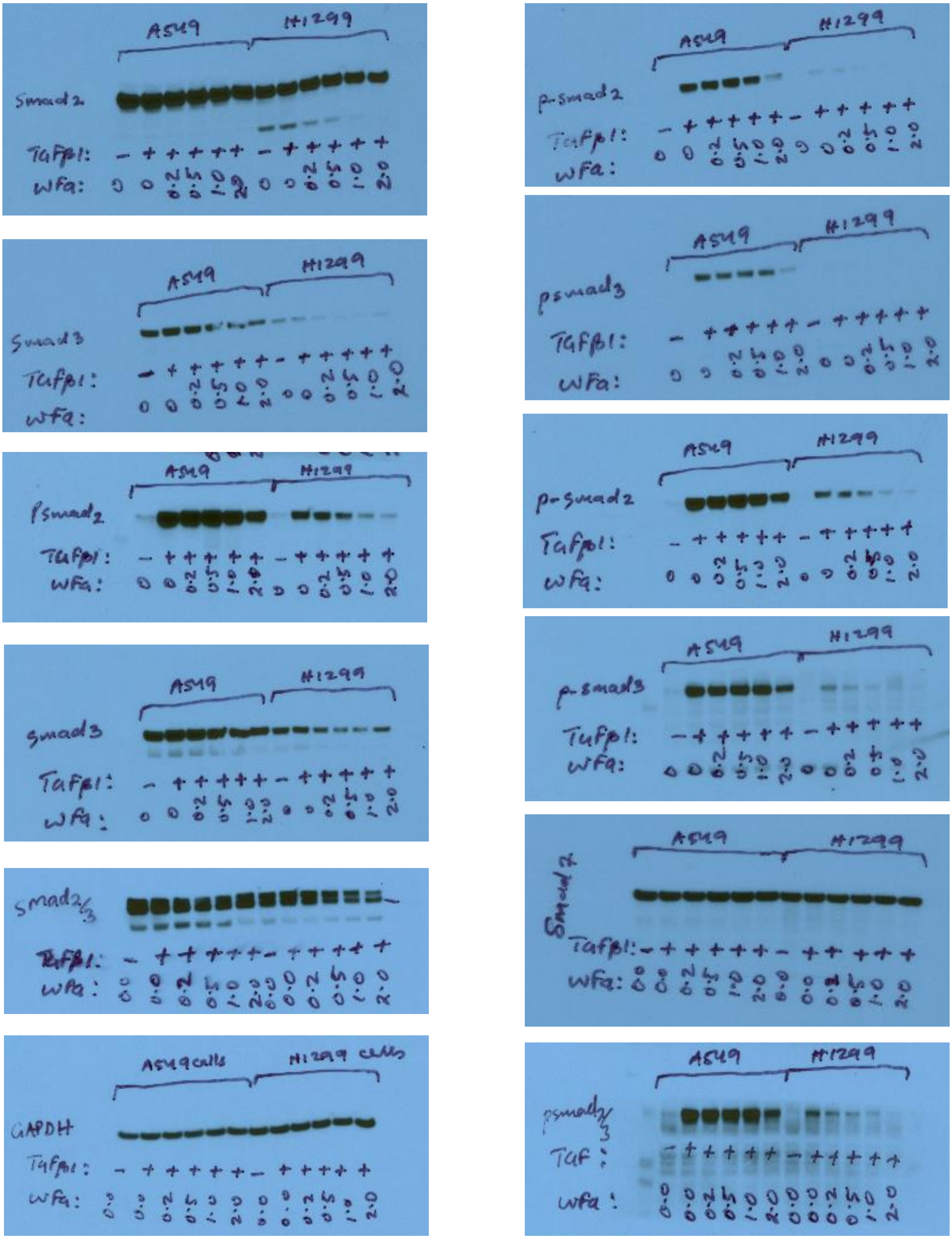

Supplementary Figure S4. Full-length unedited images of gels related to Figure 5B. Most or all the data in this supplementary figure are part of Figure 5B.

Nuclear lysates

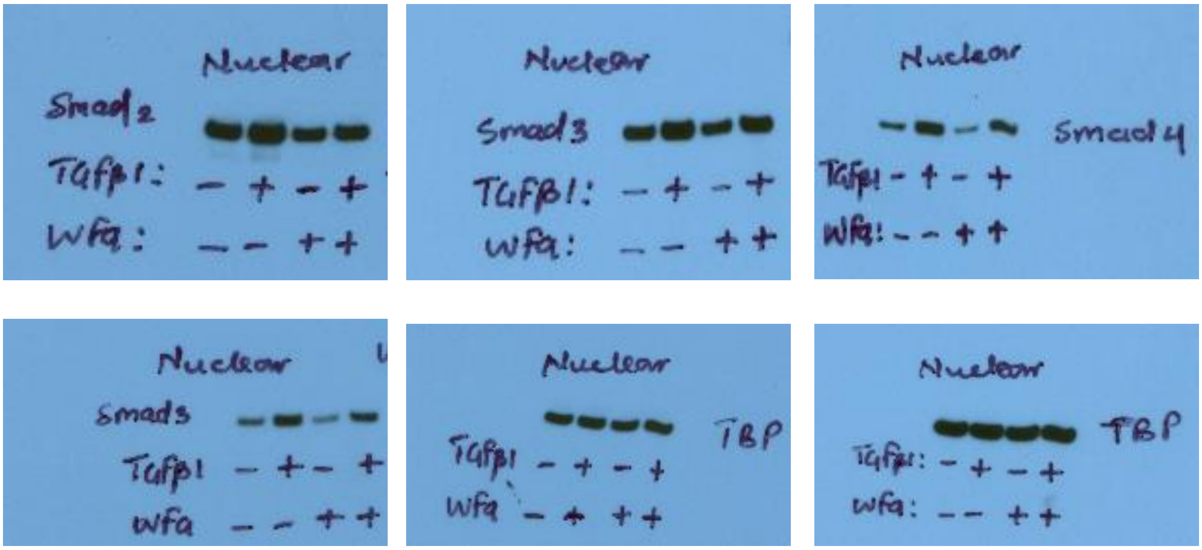

Cytosolic Fractions

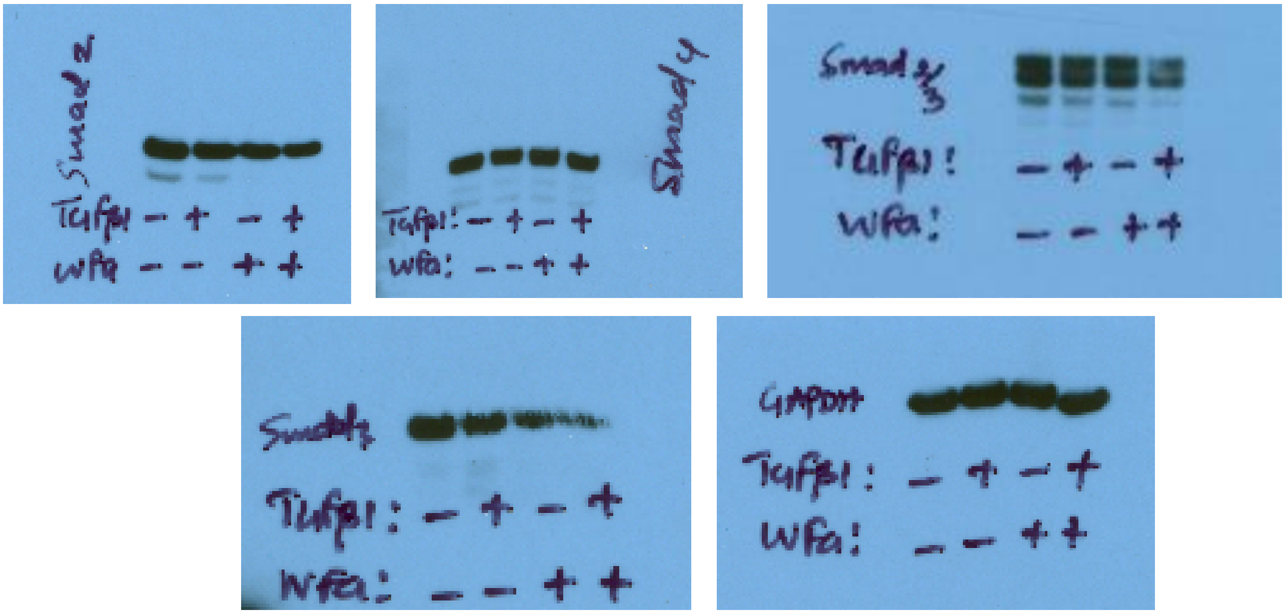

Supplementary Figure S5. Full-length unedited images of gels related to Figure 5C. Most or all the data in this supplementary figure are part of Figure 5C.

Whole cell lysate

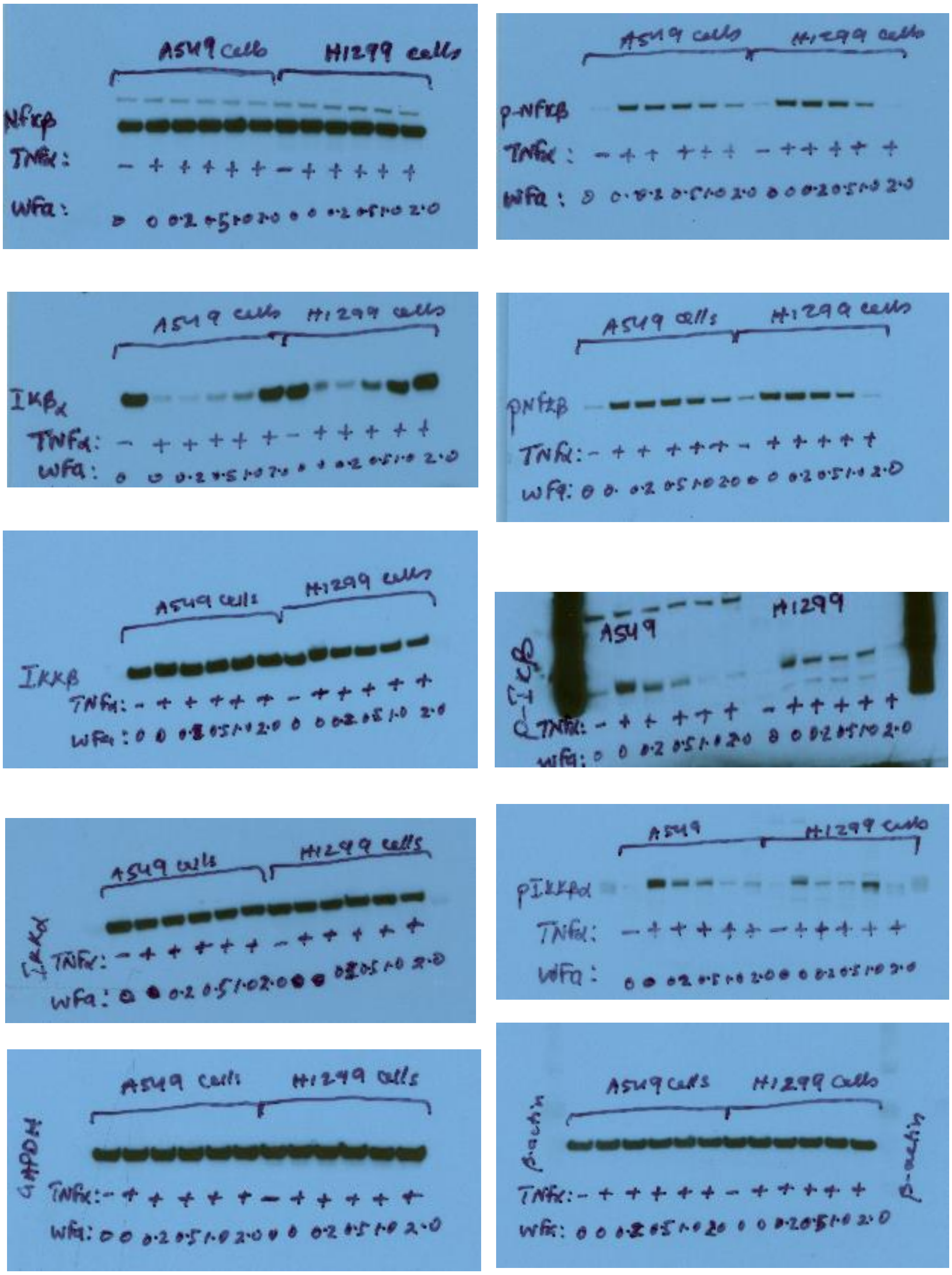

Cytosolic lysate

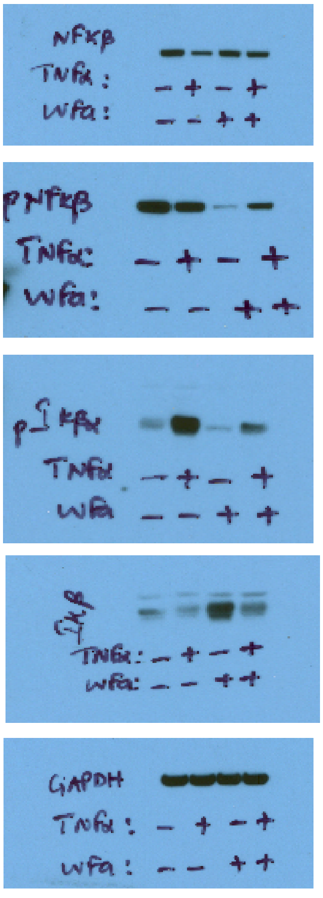

Nuclear lysate

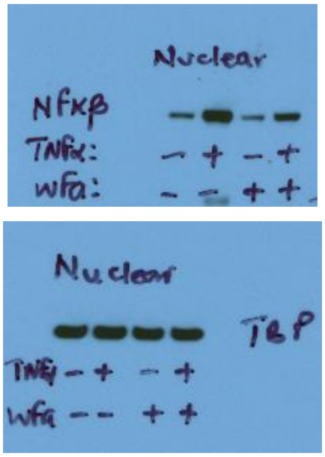

Supplementary Figure S6. Full-length unedited images of gels related to Figure 6B-C. Most or all the data in this supplementary figure are part of Figure 6B-C.

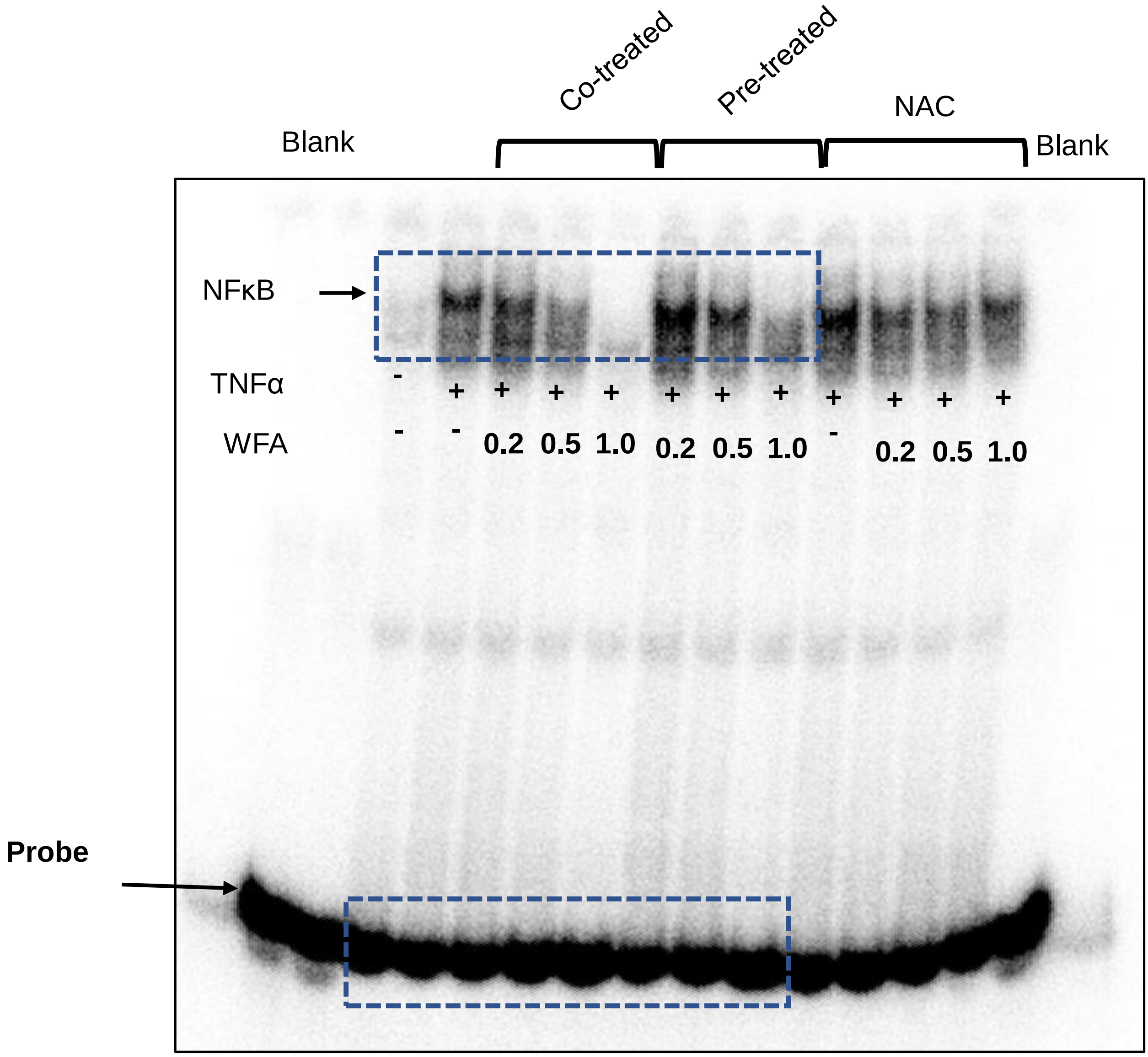

Supplementary Figure S7 Full-length unedited image of EMSA gels related to Figure 6D. The cropped image is indicated in the blue dotted area. A549 cells were either pre-treated or co-treated with indicated concentrations of WFA and stimulated with 25 ng/mL TNFα in serum-free media for 30 minutes. Additionally, cells were co-treated with WFA and TNFα in the presence of N-Acetyl Cysteine (NAC). The cells were collected by trypsinization, nuclear lysates were prepared and the DNA binding capability of NF-kB was assessed by EMSA.

Supplementary Table S1a. Band intensity Figure 3A: A549 cells (Normalized to GAPDH)

**Bands**	**1**	**2**	**3**	**4**	**5**
E-cadherin	1	0.2	0.4	0.01	0.8
N-cadherin	1	4	3	6	1.5
Snail	1	8	1.1	7.9	0.8
Vimentin	1	1.8	0.8	1.1	0.5
Fibronectin	1	1.5	0.2	5	1.3
Clauidin-1	1	0.1	1.8	1.5	0.4

Supplementary Table S1b. Band intensity Figure 3A: H1299 cells (Normalized to GAPDH)

**Bands**	1	2	3	4	5
E-cadherin					
N-cadherin	1	1.5	1.1	2.1	1.4
Snail	1	4.2	2	3.8	0.7
Vimentin	1	1.2	0.9	0.85	0.81
Fibronectin					
Clauidin-1	1	0.4	10	8.46	3.2

Supplementary Table S1c. qRT-PCR Mean CT values – Related to Figure 3B

**Group**	**GAPDH Mean CT**	**Beta actin Mean CT value**
Vehicle	16.48203087	15.72822571
TGFβ	16.50045204	15.76068783
TNFα	16.6547184	15.14597702
TGFβ1 + TNFα	16.04142189	15.51629162
TGFβ1+TNFα+WFA	16.83882713	15.41293335

Supplementary Table S2a: Relative Band intensity Figure 5A: A549 cells (Normalized to GAPDH)

Bands	1	2	3	4	5	6
Smad2	1	1.2	1.1	1.1	1.01	0.98
p-Smad2	1	21	21.1	17.8	16.3	5.4
Smad3	1	1.3	1.1	1.1	0.94	0.81
p-Smad3	1	15.3	10.8	11.1	9.75	3.2
Smad2/3	1	0.97	0.91	0.8	0.9	1.12
P-Smad2/3	1	21	19.2	19.7	17.9	5.6
Smad7	1	0.94	0.98	1.1	1.03	0.98

Supplementary Table S2b: Relative Band intensity Figure 5A: H1299 cells (Normalized to GAPDH)

Bands	1	2	3	4	5	6
Smad2	1	1.01	1.1	1.02	1.01	0.98
p-Smad2	1	10	9.5	3.8	1.8	0.9
Smad3	1	0.97	0.81	0.53	0.44	0.6
p-Smad3	1	8.1	6.2	3.01	4	1.97
Smad2/3	1	1.3	1.1	0.94	0.74	0.61
P-Smad2/3	1	5.4	3.02	1.78	1.1	0.76
Smad7	1	1.1	0.98	1.1	1.01	1.02

Supplementary Table S2c: Relative band intensity in Figure 5B – Nuclear lysate (Normalized to TBP)

Bands	1	2	3	4
Smad2	1	2.1	0.7	0.9
Smad3	1	3	0.61	1.3
Smad4	1	2.3	0.53	1.75

Supplementary Table S2d: Relative band intensity in Figure 5C – Cytosolic lysate (Normalized to GAPDH)

Bands	1	2	3	4
Smad2	1	1.01	0.92	0.84
Smad3	1	0.8	0.61	0.41
Smad4	1	0.81	0.98	0.9

Table S3a: Relative Band intensity Figure 6A: A549 cells (Normalized to GAPDH)

Bands	1	2	3	4	5	6
NF-kB	1	1.1	1.3	1.01	1.13	1.01
p-NF-kB	1	6.11	5.1	4.03	3.2	1.5
IκBα	1	0.01	0.012	0.24	0.46	1.12
p-IκBα	1	10.1	4.01	1.8	0.72	0.81
IKKβ	1	0.987	1.01	1.1	0.99	0.94
IKKα	1	1.2	1.01	1.1	1.3	1.01
p-IKKα/β	1	9.4	4.6	2.6	1.74	1.1

Table S3b: Relative Band intensity Figure 6A: H1299 cells (Normalized to GAPDH)

Bands	1	2	3	4	5	6
NF-kB	1	0.99	1.01	1.1	1	0.98
p-NF-kB	1	6.3	5.6	3.2	2.01	1.1
IκBα	1	0.1	0.2	0.31	0.61	1.1
p-IκBα	1	5	3.1	2	1.8	1.1
IKKβ	1	0.98	0.95	1.1	1.1	0.95
IKKα	1	0.98	0.943	0.87	0.79	0.81
p-IKKα/β	1	4	2.3	2.01	3.98	1.12

Table S3c: Relative band intensity in Figure 6B - Nuclear lysate (Normalized to TBP)

Bands	1	2	3	4
NF-κB	1	5.6	0.91	1.81

Table S3d: Relative band intensity in Figure 6B – Cytosolic lysate (Normalized to GAPDH)

Bands	1	2	3	4
NF-κB	1	0.48	0.87	0.777
p-NF-κB	1	0.78	0.01	0.38
IκBα	1	0.71	7.1	5.12
p-IκBα	1	8.2	0.41	1.42

Supplemental Table S4. List of antibodies used for western blot analysis

**Antibody**	**Company**	**Product Number**	**MW**	**Dilution**	**Host**
Smad2	Cell Signaling	5539	60 KDa	1:1000	Rabbit
Smad3	Cell Signaling	9523	52 KDa	1:1000	Rabbit
Smad4	Cell Signaling	38454	70 KDa	1:1000	Rabbit
Smad7	Santa Cruz	Sc-365845	46 KDa	1:1000	Mouse
Smad2/3	Cell Signaling	8685	52-60 KDa	1:1000	Rabbit
Phospho-Smad2	Cell Signaling	3108	60 KDa	1:1000	Rabbit
Phospho-Smad3	Cell Signaling	9520	52 KDa	1:1000	Rabbit
Phospho-Smad2/3	Cell Signaling	9510	52-60 KDa	1:1000	Rabbit
NFκB P65	Cell Signaling	8242	65 KDa	1:1000	Rabbit
IκBα	Cell Signaling	4814	39 KDa	1:1000	Mouse
IKKβ	Cell Signaling	8943	87 KDa	1:1000	Rabbit
IKKα	Cell Signaling	11930	85 KDa	1:1000	Rabbit
Phospho-NFκB P65	Cell Signaling	3033	65 KDa	1:1000	Rabbit
Phospho-IκBα	Cell Signaling	2859	40 KDa	1:1000	Rabbit
Phospho-IKKα/β	Cell Signaling	2697	85-87 KDa	1:1000	Rabbit
E-cadherin	Cell Signaling	3195	135 KDa	1:1000	Rabbit
Vimentin	Cell Signaling	5741	57 KDa	1:1000	Rabbit
Snail	Cell Signaling	3879	29 KDa	1:1000	Rabbit
N-cadherin	Cell Signaling	13116	140 KDa	1:1000	Rabbit
Claudin-1	Cell Signaling	13255	20 KDa	1:1000	Rabbit
Fibronectin	Santa Cruz	Sc-18825	220 KDa	1:1000	Mouse
GAPDH	Cell Signaling	5174	37 KDa	1:1000	Rabbit
Beta actin	Sigma	A3854	42 KDa	1:3000	Mouse
TBP	Millipore	MAB3658	42 KDa	1:1000	Mouse

Supplemental Table S5. List of primer sequence used for RT-PCR

Genes	Forward primer	Reverse primer
E-cadherin	5'-CCACCAAAGTCACGCTGAAT-3'	5'-GGAGTTGGGAAATGTGAGC-3'
Vimentin	5'-GAGAACTTTGCCGTTGAAGC-3'	5'-CTCAATGTCAAGGGCCATCT-3'
Snail	5'-CAGCGAGCTGCAGGACTCTA-3'	5'-GTGGGATGGCTGCCAGC-3'
Fibronectin	5'-GAGCTATTCCCTGCACCTGA-3'	5'-CGTGCAAGGCAACCACACT-3'
Beta-Actin	5'-CGTCATACTCCTGCTTGCTG-3'	5'-GTACGCCAACACAGTGCT-3'
GAPDH	5'-GACAGTCAGCCGCATCTTC-3'	5'-CAACAATATCCACTTTACCAG-3'
